# Chemical enhanced oil recovery and the dilemma of more and cleaner energy

**DOI:** 10.1038/s41598-020-80369-z

**Published:** 2021-01-12

**Authors:** Rouhi Farajzadeh, Siavash Kahrobaei, Ali Akbari Eftekhari, Rifaat A. Mjeni, Diederik Boersma, Johannes Bruining

**Affiliations:** 1 Delft University of Technology, Delft, The Netherlands; 2 Shell Global Solutions International, Amsterdam, The Netherlands; 3 Technical University of Denmark, Kongens Lyngby, Denmark; 4 Petroleum Development Oman, Muscat, Sultanate of Oman

**Keywords:** Crude oil, Environmental impact

## Abstract

A method based on the concept of exergy-return on exergy-investment is developed to determine the energy efficiency and CO_2_ intensity of polymer and surfactant enhanced oil recovery techniques. Exergy is the useful work obtained from a system at a given thermodynamics state. The main exergy investment in oil recovery by water injection is related to the circulation of water required to produce oil. At water cuts (water fraction in the total liquid produced) greater than 90%, more than 70% of the total invested energy is spent on injection and lift pumps, resulting in large CO_2_ intensity for the produced oil. It is shown that injection of polymer with or without surfactant can considerably reduce CO_2_ intensity of the mature waterflood projects by decreasing the volume of produced water and the exergy investment associated with its circulation. In the field examples considered in this paper, a barrel of oil produced by injection of polymer has 2–5 times less CO_2_ intensity compared to the baseline waterflood oil. Due to large manufacturing exergy of the synthetic polymers and surfactants, in some cases, the unit exergy investment for production of oil could be larger than that of the waterflooding. It is asserted that polymer injection into reservoirs with large water cut can be a solution for two major challenges of the energy transition period: (1) meet the global energy demand via an increase in oil recovery and (2) reduce the CO_2_ intensity of oil production (more and cleaner energy).

## Introduction

With the assumption of no breakthrough in the development of renewable energy sources, a large fraction of the global energy demand in the next few decades will be supplied by (1a) hydrocarbon fuels, (1b) natural gas^1^, (2) nuclear energy^2^, and (3) hydroelectricity^3^. Improved wind, geothermal, and solar renewable energy (currently accounting for 3.6% of the world energy consumption in regions of moderate insolation are only expected to give a small contribution to the near future energy demand, because of their relatively higher cost, insufficient investments, and lack of infrastructure^4^.

Hydrocarbon fuels generate large amounts of carbon dioxide upon burning and therefore are a major contributor to the anthropogenic climate change. Moreover, with the rise in the population of the world, more energy will be required to sustain economic growth. Then, the oil and gas industry must come up with solutions to meet the (ever)-increasing energy demand more sustainably and cleanly, while renewable sources are made more accessible and affordable. A significant portion of the produced hydrocarbon energy is consumed for fluid handling, injection of fluids, water treatment, refining, and its production, depending on the recovery mechanism^5,6^. In practice, towards the end of field lifetime, the energy required for fluid circulation may exceed the energy obtained from hydrocarbons^5,7^.

While the discovery of significant new oil and gas fields becomes less frequent, the efficient extraction of oil from existing fields, particularly with enhanced oil recovery (EOR) methods, has become essential in meeting the global energy demand^8,9^. EOR techniques are commonly applied when the natural energy of an oil reservoir or the (secondary) water injection cannot effectively produce oil^8^. The efficiency of water injection decreases when the mobility of the injected water is larger than that of the in-situ oil^10,11^. To overcome this shortcoming, water-soluble polymer molecules are added to the injected water to increase its viscosity, which reduces its mobility and thus improves the efficiency of the displacement process^8,12^. To extract the remaining oil trapped by the capillary forces, surfactants are sometimes added to lower the interfacial tension between oil and water^8^. In a typical (surfactant) polymer EOR project, injection of chemicals starts when water cut (water fraction in the total liquid produced) in the producers reaches a large value (referred to as tertiary recovery), although earlier injection of chemicals can be more efficient^8,11,13^. Injection of appropriate chemicals (under tertiary mode) generally results in reduction of water cut and increase of oil cut and hence the ultimate oil recovery.

**Figure 1:**
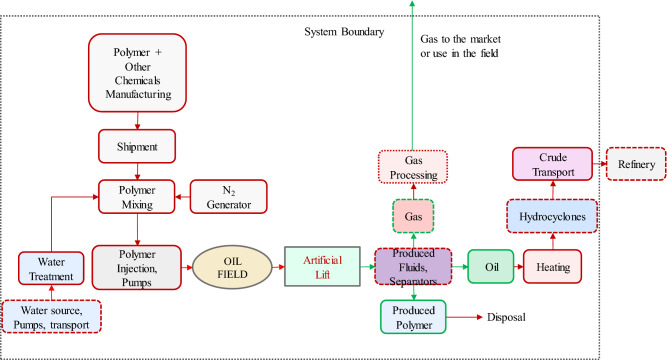
Schematic of the production cycle system and the selected boundary considered in this work for the production of oil by the injection of chemicals like polymer and surfactant. The boxes with the broken lines are either not considered in the calculations or assumed to have a negligible impact on the outcome. The red arrows are for the components with only exergy investment, while the green arrows contain part of the gained exergy.

There is a direct correlation between the CO_2_ intensity of the oil production by water injection and field water cut. Above water cuts of 90%, a large fraction of the energy obtained from oil is used in handling the injected and produced water, which leads to large amounts of CO_2_ emission^7,14^. The wide application of water injection means that reducing the carbon intensity of aging or mature oilfields with large water cuts is an essential step towards cleaner or low-carbon production of hydrocarbons^15^. This study examines the potential life-cycle impact of injecting polymer and surfactant into hydrocarbon reservoirs by considering the energy requirements of the processes. The primary focus of this study is to explore techniques to reduce CO_2_ emissions from the mature oil fields and consequently, other environmental impacts of these processes are not discussed here. We develop a method based on the concept of exergy-return on exergy-investment (ERoEI) to determine the energy efficiency and the CO_2_ intensity of polymer and surfactant EOR^16,17^. This integrated approach considers the main surface and subsurface elements of the chemical EOR process (see Fig. 1). Such analysis provides information on the energy consumption (and CO_2_ intensity) of each component, which can then be used to optimize the whole system from the energy-efficiency perspective. Moreover, energy requirements for the manufacturing of commonly used synthetic polymers, i.e., the hydrolyzed polyacrylamide (HPAM) polymer (and its CO_2_ intensity), is calculated. Our approach differs from the conventional life cycle assessment or economic analyses in that we do not rely merely on the collected production data that are often error-prone and hard to cross-check. Instead, we offer a workflow that is constructed on the laws of thermodynamics and validated mathematical models for both the reservoir and the production equipment. Our workflow facilitates the estimation of CO_2_ emission and its sensitivity to different operating parameters. Another advantage of the proposed workflow over economic analysis is that our model is based on the fundamental laws of physics and therefore will always remain valid (while the economic laws are constantly changing).

Exergy analysis is an excellent tool for quantifying the materials and energy streams and the natural limits (second law efficiency) of the energy conversion processes^18,19^. However, for quantifying the “real” embodied exergy of process equipment, e.g., a pump, it often relies on the embodied and manufacturing exergy, e.g., steel, plastic, and other material that goes into a pump and the machining of the raw material. The estimated exergy values with this approach often leads to an underestimation of the exergy value of equipment, i.e. the price per unit exergy of equipment is too high, which is not in agreement with the price per unit exergy of the fossil fuels. This does not cause large errors in the processes with large exergy streams (such as conventional oil production methods) but can introduce significant errors as soon as the recovery factors become sensitive to the equipment exergy^19^. For instance, production of unconventional oil and gas resources from the tight formations requires drilling of many wells. Drilling is an expensive technology with relatively low direct exergy consumption. The exergy consumption of the drilling can be adjusted based on the price per unit exergy of the fossil fuels or the indirect exergy consumption of the drilling need to be carefully investigated and quantified.

The structure of the paper is as follows. First, we explain different stages of the chemical EOR projects and define the system and its boundary for the assessment. Next, we provide the details of the exergy calculations followed by a brief description of the method employed to forecast the amount of the oil produced by polymer and surfactant for the reservoir of interest. Afterwards, the results of the analysis are explained as to the impact of different parameters on the exergy recovery factor. We also include real data and ensuing analyses for two reservoirs in the Middle East in which polymer is injected to improve the oil recovery. We end the paper with concluding remarks.

## System definition

Figure 1 depicts the main components of a chemical-EOR project. The analysis considers the exergy (material and process) required to manufacture the chemical (polymer and/or surfactant) and its transportation (in powder form and in large bags) to the project site. The distance between the chemical manufacturer and project site is assumed to be 7000 km. The chemicals are mixed with the treated water to prepare the injection solution with the desired viscosity. The energy consumption for the mixing is assumed to be negligible. The produced oil and gas are the exergy sources, which are shown by green arrows. The produced water initially does not contain the injected chemical; however, with time increasing amounts of chemicals are produced. The produced water (with or without chemicals) should be treated before re-injection or disposal. For the surfactant-polymer injection case, the treatment unit consists of a softening unit to remove the divalent cations. Generally, in chemical-EOR projects more energy is consumed at the production side to treat the produced (viscous) water, break the possible emulsions in the effluent, remove the chemicals from oil, etc.^8,9^. This is accounted for by assuming an additional 20% exergy requirements for produced fluids in the calculations (vs 10% in water injection). It is also assumed that 20% of the injected water is not back-produced or lost/consumed during the process. The liquids are assumed to be lifted using pumps in the producers. If the reservoirs contain viscous oil (like this study), the produced oil is heated to a certain temperature before it is transferred to a hydrocyclone, where water and other dense components are removed^20^. Finally, the oil is pumped to refineries to produce the final product, i.e., fuel.

## Exergy streams

The exergy analysis of the system defined in Fig. 1 is performed by considering the material (shown by green arrows) and work (red arrows) streams. For the calculation of exergy of the material and energy streams, we use a dead state of T_00_ = 25 °C and p_00_ = 1 atm. We also take the standard chemical exergy of elements from the work of Ref.^18^.

### Material stream

The main source of exergy is the produced oil and gas. In the case considered here the amount of produced gas is negligible. The produced oil is assumed to have a specific exergetic value of ${Ex}_{oil}^{ch}=45.63$ MJ/kg^7,14^. The exergy of the produced water (with and without chemicals) is assumed to be negligible.

### Work streams

Table 1 provides the relations used to calculate the exergy rate of the system components. The details of the calculations for the processes common with the waterflooding (water treatment, pumping and lifting the liquids, heating, transportation, etc.) can be found in Ref.^7^. The overall pump efficiency, $\eta$ is assumed to be 36%^16^.

**Table 1:** Summary of the required exergy for material and work streams.

Material stream	Specific exergy (MJ/kg)	Work stream	Specific exergy
Crude oil	45.63	Injection pump (${\dot{Ex}}_{liquid}^{pr,pump})$	$\dot{Q}\Delta P/\eta$ (J/s)
Gas (methane)	52	Artificial lift (${\dot{Ex}}_{liq}^{th,lift})$	[$\dot{Q}\left({f}_{w}{\rho }_{w}+\left(1-{f}_{w}\right){\rho }_{o}\right)gh]/\eta$ (J/s)
Produced water	0.0	Water treatment	18 (kJ/kg) (5 kWh/m^3^)^21^
		Heating of crude oil (${\dot{Ex}}_{oil}^{heating})$	${\dot{m}}_{oil}{c}_{p}\Delta T$ (J/s)
		Transport of oil to refinery	188 J/kg-km^22^
		Other process	20% (10%) of the total exergy for polymer/surfactant (water)
		Polymer manufacturing	123,600 (kJ/kg)
		Surfactant manufacturing	62000^23^ (kJ/kg)
		Water softening	50^24^ (kJ/kg)

### Polymer manufacturing

Several polymer types are used in polymer flooding; however, polyacrylamides are the most widely-used polymer types in EOR projects because of their low cost^8,9^. Polyacrylamides undergo hydrolysis (with the degree of hydrolysis depending on the conditions) and are therefore called hydrolyzed polyacrylamides or HPAMs. Polyacrylamides are produced by polymerization of acrylamides, which in turn are obtained via catalytic hydration of acrylonitrile. The practical exergy of polyacrylamide is calculated by adding the cumulative exergy consumption of each material multiplied by the amount required for the production of one tonne of polymer. The details of the procedure are explained in Tables 3 and 4 in Appendix 1. The practical exergy for manufacturing of the HPAM polymers is calculated to be 123.6 MJ/kg-polymer. The CO_2_ emission is estimated by calculating the CO_2_ emission of each material stream, which is done by multiplying the fraction of the cumulative exergy consumption originating from fossil fuels by the specific CO_2_ emission of the fuel, i.e., natural gas, oil and coal with respective estimated specific CO_2_ emission of 0.055, 073, and 0.088 kg-CO_2_/MJ^17^. This results in CO_2_ emission of 3.25 (gas), 4.72 (oil), and 6.35 (coal) kg-CO_2_ per kg-polymer, depending on the type of consumed fossil fuel.

### Surfactant manufacturing

Schowanck et al.^23^ provide life-cycle assessment and greenhouse-gas intensity for production of the common surfactants used in the European detergents and personal care products using the data from 16 major surfactant manufacturers. The linear alkylbenzene sulphonic acid is considered as the proxy for the EOR surfactants. The total energy consumption for these surfactants is 62 MJ/kg-surfactant, and their manufacturing results in a total CO_2_ emission of 1.75 kg CO_2_ per kg surfactant^23^. In the manufacturing of these surfactants, different sources of primary energy from renewable and fossil fuels are used (although the contribution of renewables is not significant). The CO_2_ emissions from the fossil fuels used in the manufacturing process is about 1.56 kg CO_2_/kg-surfactant. The reported numbers are the average values for several surfactant-manufacturing companies.

### Exergy recovery factor

The rate exergy recovery factor, $E{x}_{RF}$, is a measure of the sustainability of the system and is defined as the ratio between the net and gross exergy gains, i.e.,

1 $$E{x}_{RF}=\frac{E{x}_{gained}-E{x}_{invested}}{E{x}_{fuel}}$$

For the production of oil by injection of chemicals, Eq. (1) can be re-written as

2 $$E{x}_{RF}=1-\frac{{E{x}_{chemicals}^{manufacturing}+Ex}_{water}^{pr,pump}+{Ex}_{fluid}^{pr,lift}+{Ex}_{oil}^{pr,trans}+{Ex}_{water}^{pr,treatment}{+Ex}_{oil}^{pr,heating}+{Ex}^{pr,other}}{E{x}_{oil}^{ch}+E{x}_{C{H}_{4}}^{ch}}$$

The cumulative exergy recovery factor is calculated from

3 $$E{xc}_{RF}=\frac{{\int }_{0}^{t}(E{x}_{gained}-E{x}_{invested)dt}}{{\int }_{0}^{t}E{x}_{fuel}dt}$$

## Production forecast

A synthetic reservoir model, called “Egg Model”, with the properties summarized in Table 2 is used to generate the oil-recovery histories for water, polymer, and surfactant-polymer injection. This geological model has four producers and eight injectors. The reservoir has a constant porosity of 20% and varying permeability of 80–7000 mD. The reservoir initially contains 8.65 × 10^5^ m^3^ of oil. More details on the geological model can be found in Refs.^14,25^. Each grid block is 8 by 8 m with a height of 4 m. The initial pressure of the reservoir is set to 90 bar. The producers operate at a bottomhole pressure of 65 bar and the injectors have a rate constraint of 79.5 m^3^/day/well with a maximum allowable pressure of 140 bar.

**Table 2:** Reservoir and fluid properties for the base case.

Symbol	Variable	Value	Unit
*Φ*	Porosity	0.2	–
*c*_*w*_	Water compressibility	1.0 × 10^–10^	/Pa
μ_o_	Oil viscosity	0.10	Pa s
*μ*_*w*_	Water viscosity	6.5 × 10^–4^	Pa s
*k*^*0*^_*ro*_	End-point relative permeability, oil	1.0	–
*k*^*0*^_*rw*_	End-point relative permeability, water	0.5	–
*n*_*o*_	Corey exponent, oil	1.8	–
*n*_*w*_	Corey exponent, water	2.7	–
*S*_*or*_	Residual oil saturation	0.3	–
*S*_*wc*_	Connate-water saturation	0.09	–
*P*_*init*_	Reservoir initial pressure	90	bar
*OIIP*	Oil volume initially in the reservoir	8.65 × 10^5^	m^3^

In the polymer injection (PI) case, a polymer solution with a viscosity of 15cP (concentration of 1200 ppm) is injected when the overall water cut in the field reaches 90%. In the surfactant-polymer injection (SP) case, when the water cut reaches the value of 90%, 0.30 pore volume of surfactant-polymer mixture [with surfactant concentration of 3000 ppm and polymer viscosity (concentration) of 50cP (2800 ppm)] is injected into the reservoir. The brine used for preparing the surfactant solution is soft water, i.e., contains no divalent ions. This process is followed by injection of a polymer-only chase with viscosity of 50cP (concentration of 1800 ppm). In the design of this process, due to the presence of the alkali in the surfactant-polymer slug, more polymer concentration is required to obtain a viscosity of 50cP^8,12^.

## Results and discussion

**Figure 2:**
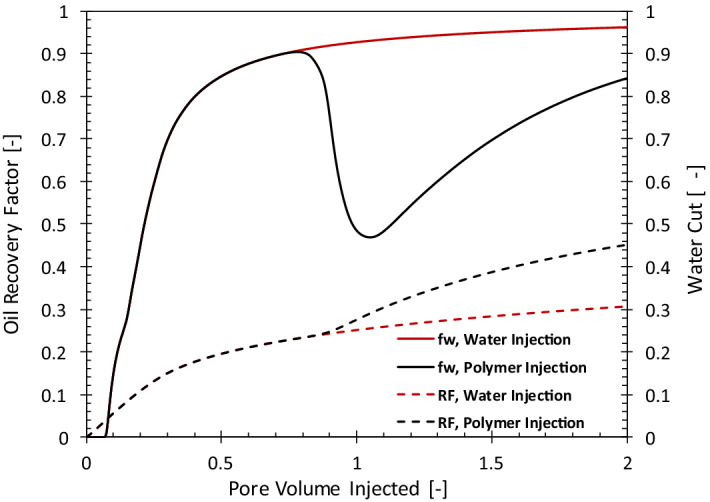
The oil recovery factor (RF) and water cut (*f*_*w*_) histories for water and polymer injection.

The output of the numerical simulations provides the amounts of injected water, pressure drop, injected chemicals as well as the produced oil and water. These numbers are used as input to perform the exergy analysis. As an example, Fig. 2 shows the simulated oil-recovery-factor and water-cut (*f*_*w*_) histories for the polymer- and water-injection cases. The oil recovery factor (dashed line) is defined as the volumetric ratio of cumulative produced oil and the initial oil in the reservoir. In the water-injection (WI) case, the water cut (the ratio between the produced water and total liquid production) keeps increasing because oil production decreases with time. After about two pore volumes of water injection, the water cut reaches the value of 96%.

**Figure 3:**
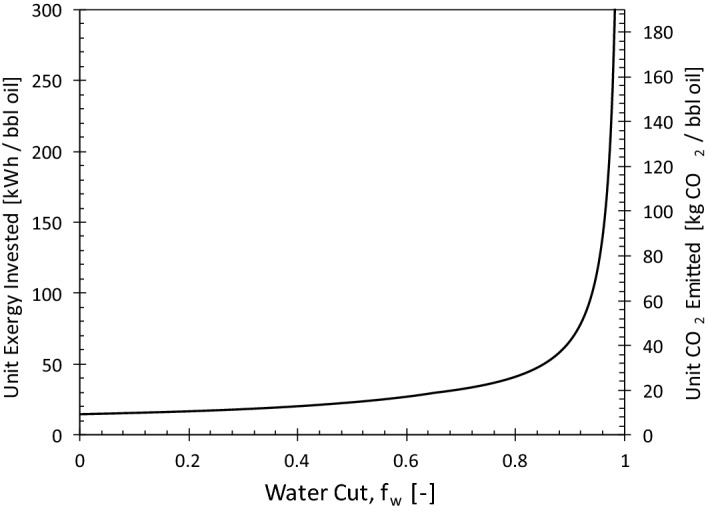
Unit exergy consumed and CO_2_ emitted as functions of water cut for the water-injection case.

Figure 3 shows the exergy invested to produce one barrel of oil (and its corresponding CO_2_ emission) as a function of the water cut for the water-injection case. The unit CO_2_ emission (kgCO_2_/bbl oil) is the product of the unit exergy invested and the average emission rate of the electricity production in the location of the project, which is assumed to be the Middle East (with CO_2_ intensity 650 gCO_2_/kWh)^26^. The major exergy investment in water-injection projects relates to the circulation of water^7,14^. As a result, the unit exergy investment strongly depends on the water cut. For *f*_*w*_ > 90%, a small increase in the water cut leads to significant exergy dissipation or loss due to handling of excessive amounts of water. Accordingly, at large water cuts, the unit CO_2_ emission becomes considerable. For example, from Fig. 3 for f_w_ = 96% the unit CO_2_ emission is ~ 100 kg/bbl of oil. For comparison, the specific CO_2_ emission of crude oil is about 73 gCO_2_/MJ or 3.14 kgCO_2_/kg oil. Assuming an oil density of 850 kg/m^3^, burning one barrel of oil will approximately produce 425 kg of CO_2_. This implies that the indirect CO_2_ emissions resulting from the production of oil at high water cuts could become comparable to direct CO_2_ emissions from its combustion. Therefore, by operating oil reservoirs at lower water cuts release of large amounts of CO_2_ could be avoided. The optimum operating conditions of an oil field (in terms of maximum net present value or minimum CO_2_ emission) does not necessarily lead to maximum oil production from the reservoir^14^. Such optimum operations also leave more oil in the reservoir, which can later be produced by a technique with a comparatively smaller CO_2_ footprint such as polymer flooding, as discussed next.

Injection of polymer reduces the mobility of the displacing phase and results in the reduction of water cut, as shown in Fig. 2. In the case considered here, polymer injection starts when *f*_*w*_ = 90%. The higher viscosity of the injected polymer compared to water results in more efficient extraction of oil from the reservoir; for instance, after injection of 1 PV of polymer, the recovery increases from 22 to 41%. The water cut goes through a minimum and once the polymer front reaches the producer the water cut starts to increase. Eventually, the amount of the oil produced by the polymer becomes negligible and *f*_*w*_ becomes very large.

**Figure 4:**
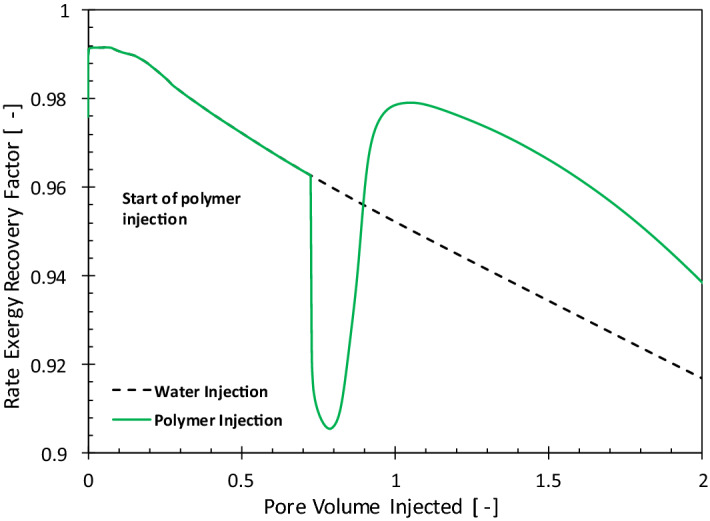
History of the rate exergy recovery factor (Ex_RF_) for polymer (solid line) and water-injection (dashed line) cases.

Figure 4 compares the history of the rate exergy recovery factor for WI and PI cases. For the WI case, the rate exergy recovery factor (Eq. 2) keeps decreasing because with time more energy is required to recover oil. After 2 PV of total injection, about 9% of the oil exergy is invested in producing it. With the injection of polymer, the exergy recovery factor suddenly decreases, because at this stage the exergy invested for manufacturing and injecting polymer is larger than the exergy gain from oil. However, once the oil bank created by the injected polymer solution arrives at the producers, the exergy gain from oil surpasses the exergy investment and the exergy recovery factor increases. The maximum exergy recovery factor in Fig. 4 corresponds to the minimum in the water cut for the PI case in Fig. 2, which reiterates the negative impact of circulation of large volumes of water on the energy efficiency of the oil production systems. The area enclosed between the dashed line (decline curve for waterflooding) and the polymer injection is the incremental exergy gain (the area above the dashed line) or loss (area below the dashed line). The net exergy recovery is the difference between the upper area and the lower area. It is possible to get a negative net exergy recovery for polymer flooding compared to water flooding, especially when the injection of polymer does not efficiently reduce the water cut.

**Figure 5:**
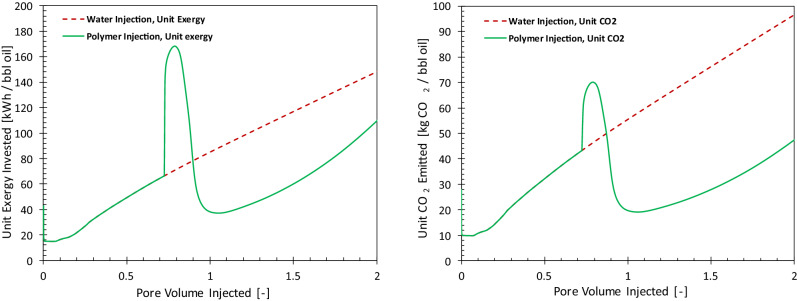
Histories of the unit energy consumed (left) and unit CO_2_ emitted (right) for water injection (dashed line) and polymer injection (solid line) cases.

**Figure 6:**
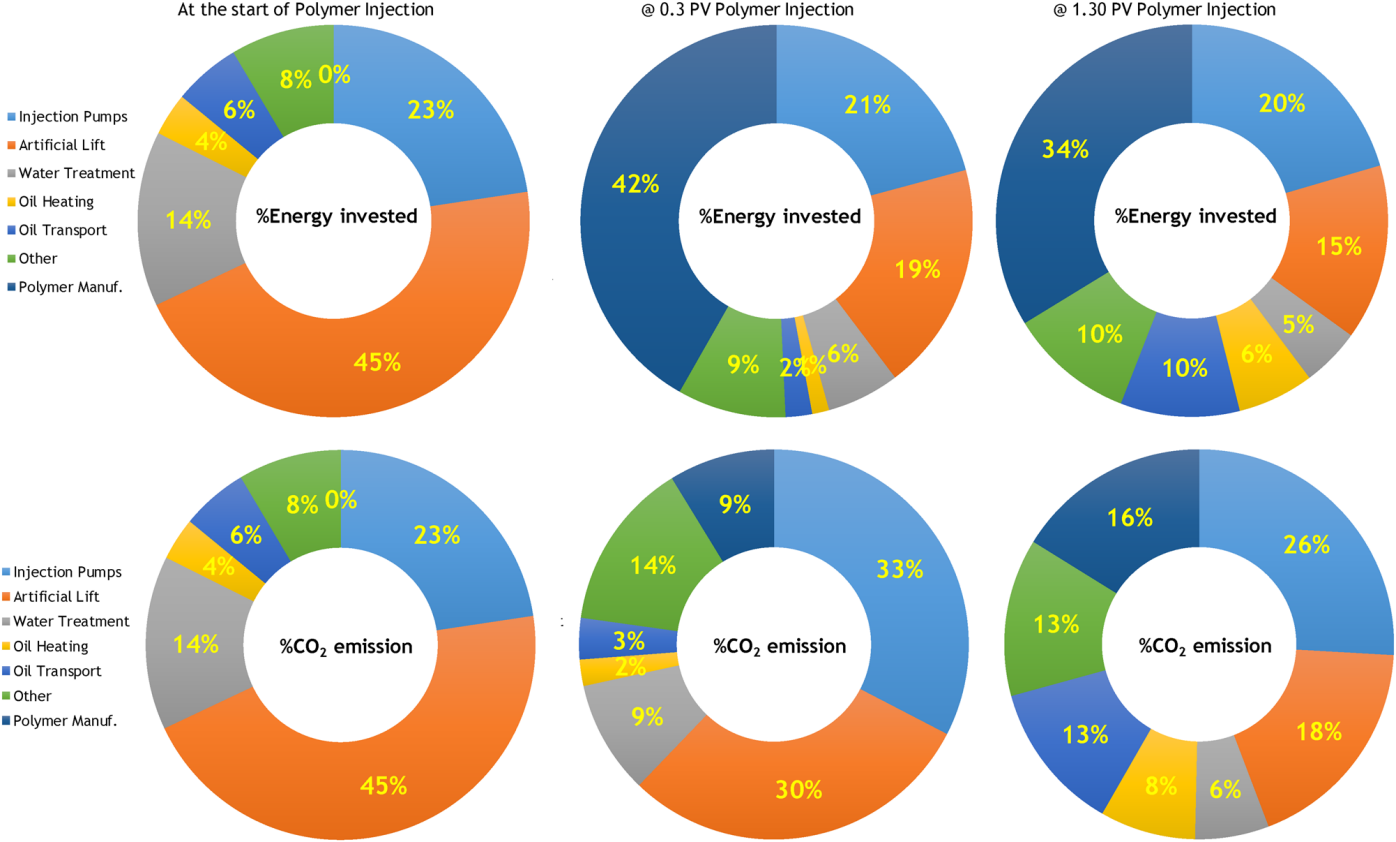
Fractions of exergy invested and CO_2_ emission at different time intervals for water and polymer injection cases.

Figure 5 compares the unit exergy invested (left) and the unit emitted CO_2_ (right) of WI (dashed red curve) and PI (solid green curve) cases. For the WI case, the unit exergy investment and the unit CO_2_ emission keeps increasing in accordance with the increase in volumes of injected and produced water. With the start of the polymer injection, the exergy investment also increases, which results in a larger unit CO_2_ emission due to exergy investments in manufacturing and shipment of the injected polymer. However, once the injection of polymer reduces the water cut, both exergy investment and CO_2_ emissions are reduced. For example, at 1 PV of total injection, the unit CO_2_ emission decreases to 20 kgCO_2_/bbl from the base case of 55 kgCO_2_/bbl. Figure 6 presents the fractions of the unit exergy invested and unit CO_2_ emitted for the PI case. At the start of the polymer injection, the energy invested on artificial lift and injection pumps comprise 68% of the exergy consumed for producing the oil. Of the other parts of the invested energy water treatment is a large contributor. A large invested exergy is reflected in a large contribution to greenhouse gas emission. Polymers act to reduce cumulative pump costs. After 0.3 PV of polymer injection, about 42% of the total exergy is invested in polymer manufacturing and shipment. However, after breakthrough of the polymer bank, exergy investment in the handling of the produced fluids increases again and the share of polymer exergy decreases. It is interesting to note that despite the large exergetic cost of the injected polymer, the related CO_2_ emission is not in direct relation with the exergy investment. For example, at 0.3 PV of polymer injection, only 9% of the total CO_2_ emission is due to the injected polymer. This is mainly because the injected polymer does not oxidize in the reservoir. It is either retained in the reservoir (via adsorption and mechanical entrapment) or degraded, and in case of production, it is disposed. Therefore, no additional CO_2_ is generated from the polymer. In other words, CO_2_ is sequestered in the form of polymer.

**Figure 7:**
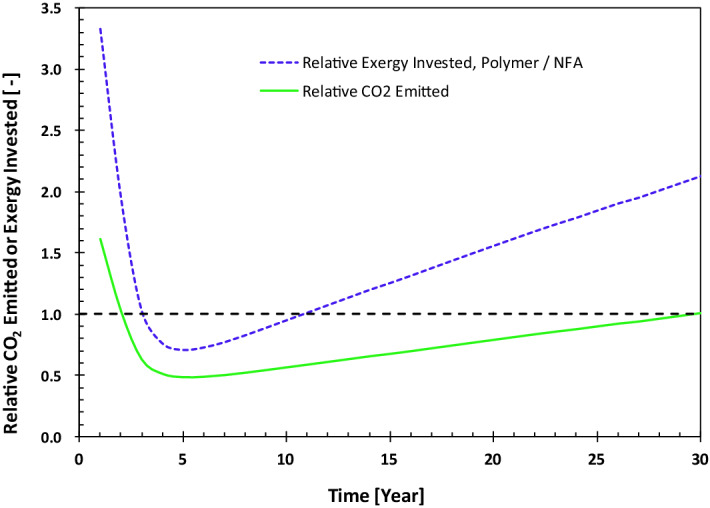
History of relative exergy invested and CO_2_ emitted for field A in the Middle East. The calculations are based on simulation results. NFA stands for no further action, i.e., production by natural aquifer drive.

**Figure 8:**
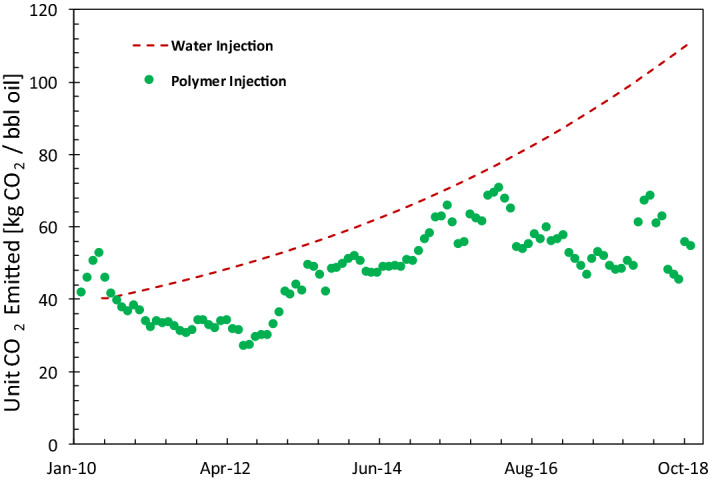
History of CO_2_ emitted for field B in the Middle East from the start of the project in January 2010 until October 2018. The calculations are based on the data from one injection pattern in the field.

Figures 7 and 8 show examples of these calculations for two fields in the Middle East. The field example in Fig. 7 is a reservoir with a strong bottom aquifer. The oil is produced by influx of water from the aquifer, while the reservoir pressure remains nearly constant due to the strength of the aquifer. This means that no additional water injection is required to displace the oil, i.e., ${\dot{Ex}}_{water}^{pr,pump}=0$. Figure 7 plots the ratios of the CO_2_ emitted and exergy invested between polymer injection and natural aquifer drive.. In this case, polymer EOR has larger exergy investment, because it requires pumping of significant volumes of polymer-containing water, i.e., ${\dot{Ex}}_{polymer}^{pr,pump}>0$. However, injection of polymer, leads to reduction in exergy investment in lift pumps and excessive water treatment. This combined with the fact that polymer does not produce CO_2_ in the reservoir (no oxidation) eventually results in smaller CO_2_ footprint for the PI case compared to the aquifer-drive production scheme.

Figure 8 uses the oil recovery results of a polymer injection pattern in another field in the Middle East. Water injection was the main recovery mechanism for this reservoir until the water cut reached the value of 95% in January 2010. At this point, the injection of polymer started, which resulted in a water-cut reversal, similar to Fig. 2. The dashed line and the circles in Fig. 8 compare the unit CO_2_ emission for water injection (in case it continued) and polymer injection. The polymer-injection project has a significantly smaller carbon footprint.

**Figure 9:**
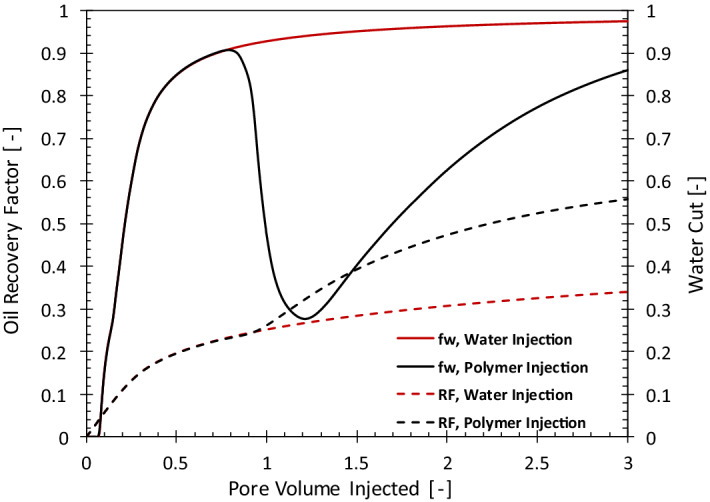
The oil recovery factor (RF) and water cut (f_w_) histories for water and surfactant-polymer injection.

**Figure 10:**
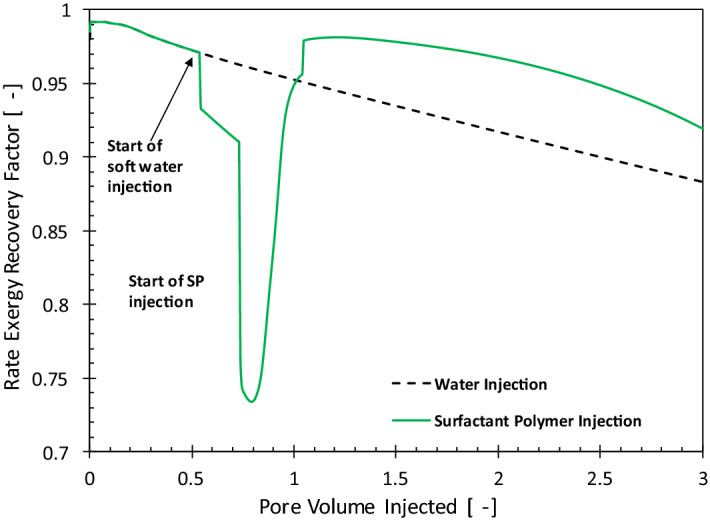
History of the rate exergy recovery factor (*Ex*_*RF*_) for surfactant-polymer (solid line) and water-injection (dashed line) cases.

Figure 9 shows the impact of injecting surfactant/polymer on oil recovery and water cut in comparison with the water injection. The characteristics of the oil recovery are similar to that of the polymer flooding, i.e., injection of surfactant/polymer leads to the formation of an oil bank. As a result, the water cut first decreases (more significantly than polymer injection) and after the breakthrough of the chemicals, the water cut starts to increase. The history of the exergy recovery factor for the surfactant-polymer injection, shown in Fig. 10, is similar to that of the polymer injection. For the surfactant-flooding, the first deviation from water flooding (dashed line) occurs when soft water is injected to remove the divalent ions. With the injection of the chemicals, the rate exergy recovery factor significantly decreases, which is because of the large exergy invested in the manufacturing of the chemicals (surfactant and polymer) compared to the small exergy gain before arrival of the oil bank in the producers. To maintain stable displacement larger viscosity (or polymer concentration) is required in surfactant/polymer injection^12^. Moreover, manufacturing of the HPAM polymer is exergetically two times more expensive than the surfactant. Therefore, the exergy recovery factor for the SP case is smaller than for the PI case. Nevertheless, injection of SP extracts more oil and consequently with the breakthrough of the oil bank the exergy recovery rises above the WI case. Similar to the PI case, the maximum in the exergy recovery factor corresponds to the minimum in the water cut.

**Figure 11:**
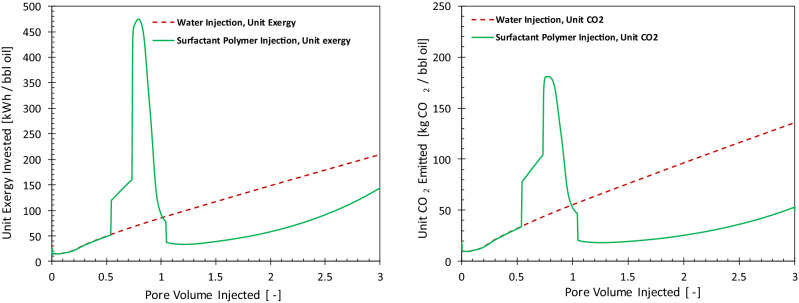
Histories of the unit energy consumed (left) and instantaneous unit CO_2_ emitted (right) for water injection (dashed line) and surfactant-polymer injection (solid line) cases.

Figure 11 presents the unit exergy invested and the calculated CO_2_ footprint of the SP case. The difference between the area enclosed above and below the water-injection case (dashed line) provides the net exergy (in the left figure) and the net CO_2_ emission (in the right figure). Even though the net unit exergy invested can be negative for the SP flooding, since the majority of the invested exergy is in the form of material it will not necessarily emit more CO_2_ than water injection at large water cuts. In the case investigated here, application of SP leads to saving of large quantities of CO_2_. For example, the continuation of water injection at 2 PV results in 95 kg/bbl of produced oil, while this number is less than 25 kg/bbl for SP flooding (similar to that of the polymer flooding). The extent of CO_2_ emission in the surfactant/polymer process depends largely on the amount of the chemicals injected. For example, if an alcohol is used in the surfactant formulation as a co-solvent, the net CO_2_ emission could be negative.

**Figure 12:**
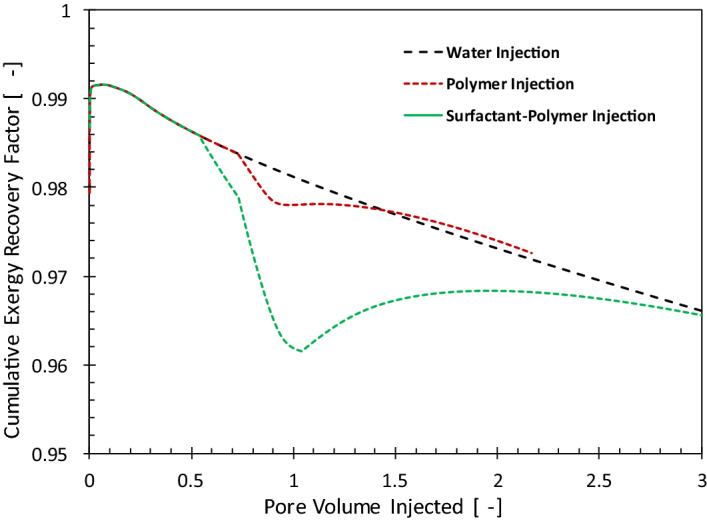
History of cumulative exergy recovery factor for water-, polymer- and surfactant/polymer-injection cases.

Figure 12 compares the cumulative exergy recovery for water-, polymer- and surfactant/polymer-injection cases. For the geological realization and injection compositions considered here, at the end of the respective projects, the cumulative exergy recovery factors of the three processes are similar. It is notable that, in the cumulative sense, only a minor fraction of the exergy gained from oil (about 3%) is invested to produce it. Furthermore, the small difference between the three curves indicates that the ultimate exergy gain from oil is considerably larger the exergy required to extract it. The additional exergy invested in manufacturing and injection of the chemicals is largely compensated by the exergy gain from the incremental oil.

## Conclusions

In this study, we develop a method based on the concept of exergy-return on exergy-investment (ERoEI) to assess the life-cycle impact of polymer and surfactant enhanced oil recovery (EOR). The following conclusions are drawn from this study.

- The exergy concept facilitates the assessment of the life-cycle efficiency of chemical enhanced oil recovery projects.
- The exergy recovery factor for chemical EOR decreases with time. This indicates that the process exergy requirements to produce the exergy increases with time.
- The practical exergy of manufacturing hydrolyzed polyacrylamide (HPAM) polymers is estimated to be 123.6 MJ/kg. This gives a CO_2_ emission of 3.25 (gas), 4.72 (oil), and 6.35 (coal) kg CO_2_/kg polymer.
- Compared to water flooding, injection of polymer generally increases the energy efficiency of the oil recovery system. Because of additional oil production (exergy gain) and smaller water circulation (exergy investment), the project time-averaged energy invested to produce one barrel of oil from polymer flooding is smaller than that of the prolonged water flooding because of handling of large water volumes.
- Exergy investment in some chemical EOR projects might be larger than that of waterflooding; however, since major fraction of the exergy investment is in the form of materials, the CO_2_ intensity of the oil produced from these projects is considerably smaller compared to water injection at large water cuts.
- Polymer injection into reservoirs with large water cut can be a solution for two major challenges of the energy transition period: (1) meet the global energy demand via an increase in oil recovery and (2) reduce the CO_2_ intensity of produced oil (more and cleaner oil).
- For surfactant EOR, the extent of improvement in energy efficiency depends on the incremental gain and quantity of the chemicals in the injected formulation. However, in many cases, the oil produced by surfactant EOR will be cleaner than the oil produced by water injection at large water cuts.

## Appendix 1

Tables 3 and 4 show the material and energy streams for the production of acrylonitrile and polyacrylamide, respectively. The exergy required for the production of acrylonitrile is estimated to be 78.9 MJ/kg^27,28^. Phosphoric acid has a chemical exergy of 980 kJ/kg. Assuming a production efficiency of 33%, a value of 3.0 MJ/kg is calculated^29,30^. However, by including all the exergy consumption in the life cycle of phosphoric acid, an exergy value of 47.65–57.65 MJ/kg is obtained, of which 31% is fossil fuel^31^. A large fraction of this value accounts for the environmental losses due to land use. For sodium hydroxide, we use a value 27.99 MJ/kg, of which 39% is fossil fuel (5). For activated carbon, which can be produced from coal or biomass, we follow Bayer et al.^32^ assuming 1600 kWh plus 12 tons of steam consumption (produced by burning 330 m^3^ of natural gas to produce 1 ton of activated carbon from coal). Assuming average exergy values of 500 kJ/mol and 800 kJ/mol, respectively, for coal (CH) and natural gas (CH_4_), the average exergy of activated carbon is estimated to be 116.9 MJ/kg. The chemical and practical exergy of copper are reported to be 0.172 MJ/kg and 66.7 MJ/kg, respectively^33^. The exergetic efficiency of hydrogen production by steam reforming process is around 70%^34^. This gives a practical exergy of 168.6 MJ/kg or 9.46 MJ/m^3^ (at standard condition) for hydrogen. Depending on the environmental condition, an exergy value of 1 kJ/kg (or 1 MJ/m^3^) is expected for the cooling water^35^. The exergy of steam is calculated based on the heat of vaporization of water, which is 2257 MJ/tonne. We round the number to 2500 MJ/ton to include a small heat loss. We also assume that the required heat is provided from the combustion of natural gas. For mineral oil, which is usually an alkane, we assume an average chemical exergy of 53.6 MJ/kg.

**Table 3:** Material and exergy required for manufacturing of acrylonitrile by catalytic hydration^36,37^.

Material	Consumption (unit/ton)	Unit	Exergy	Exergy unit	Total (MJ/ton acrylamide)
Acrylonitrile	0.7616	Tonne	78.9	MJ/kg	60,090.24
Activated carbon	0.0158	Tonne	116.9	MJ/kg	1847.02
Cu–Cr	0.0018	Tonne	66.7	MJ/kg	120.06
Hydrogen	65.5945	Nm^3^	9.46	MJ/Nm^3^	620.52397
Cooling water	159.397	m^3^	1	MJ/m^3^	159.397
Electricity	133.6	kWh	9	MJ/kWh	1202.4
Inert gas	304.171	Nm^3^	0		0
Process water	1.33526	m^3^	0		0
Steam	4.878	Tonne	2500	MJ/tonne	12,195

**Table 4:** Material and exergy streams for the production of one ton of polyacrylamide^36,37^.

Material and energy	Amount	Unit	CExC	Unit	% fuel
Acrylamide	1.0222	Tonne	76.23	MJ/kg	0.41
Caustic soda (NaOH)	0.10647	Tonne	28	MJ/kg	0.45
Mineral oil	0.6782	Tonne	53.6	MJ/kg	0.4
Phosphoric acid	0.04719	Tonne	25	MJ/kg	0.31
POE laurylether	0.06152	Tonne	53.6	MJ/kg	0.4
Sodium bisulfite	0.00016	Tonne	28	MJ/kg	0.5
Sodium bromate	0.00008	Tonne	28	MJ/kg	0.5
Sorbitol monooleate	0.06073	Tonne	–	–	1
Cooling water	458.163	m^3^	1	MJ/m^3^	1
Electricity	116.845	kWh	9	MJ/kWh	1
Steam	0.158	Tonne	2500	MJ/tonne	1

The practical exergy of polyacrylamide is simply calculated by the sum of the cumulative exergy consumption of each material multiplied by the amount that is required for the production of 1 ton of polymer. This gives a value of 123.6 MJ/kg for HPAM polymer. The CO_2_ emission is estimated by calculating the CO_2_ emission of each material stream, which is done by multiplying the fraction of the cumulative exergy consumption that comes from fossil fuels by the CO_2_ emission of a fossil fuel, i.e., natural gas with an estimated CO_2_ emission of 0.055 kg/MJ or coal with a CO_2_ emission of 0.088 kg/MJ. This gives a CO_2_ emission of 3.25 (gas), 4.72 (oil), and 6.35 (coal) kg CO_2_/kg polymer, depending on the type of consumed fossil fuel.

## Abbreviations

EOR: Enhanced oil recovery
ERoEI: Exergy-return on exergy-investment
HPAM: Hydrolyzed polyacrylamides
PI: Polymer injection
PV: Pore volume
SP: Surfactant-polymer injection
WI: Water injection
$Ex_{oil}^{ch}$: Specific chemical exergy of oil (J/kg)
$Ex_{{CH_{4} }}^{ch}$: Specific chemical exergy of methane (J/kg)
${\mathop {Ex}\limits^{ \cdot }}_{{liquid}}^{{pr,pump}}$: Exergy rate of a pump (J/s)
${\mathop {Ex}\limits^{ \cdot }}_{{liq}}^{{th,lift}}$: Exergy rate to lift the produced liquid (J/s)
${\mathop {Ex}\limits^{ \cdot }}_{{heating}}^{{oil}}$: Exergy rate for heating the crude oil (J/s)
$Ex_{RF}$: Exergy recovery factor
$Ex_{gained}$: Produced exergy corrected for material (J)
$Ex_{invested}$: Invested process exergy (J)
$Ex_{fuel}$: Gross exergy of the source (J)
$Ex_{{}}^{pr,other}$: Required exergy for other processes (J)
$Ex_{chemicals}^{manufacturing}$: Required exergy for chemical manufacturing (J)
$Ex_{water}^{pr,pump}$: Required exergy for injecting water with a pump (J)
$Ex_{fluid}^{pr,lift}$: Required exergy for lifting the produced fluid (J)
$Ex_{oil}^{pr,trans}$: Required exergy for oil transportation (J)
$Ex_{water}^{pr,treatment}$: Required exergy for treatment of produced water (J)
$Ex_{oil}^{pr,heating}$: Required exergy for heating produced crude oil (J)
*c*_*p*_: Specific energy required to heat the produced oil (J/kg/K)
*c*_*w*_: Water compressibility (/Pa)
*f*_*w*_: Water volume fraction
*g*: Gravitational acceleration (m/s^2^)
*h*: Reservoir depth (m)
*k*^*0*^_*ro*_: End-point relative permeability, oil
*k*^*0*^_*rw*_: End-point relative permeability, water
$\dot{m}_{oil}$: Oil mass rate (kg/s)
*n*_*o*_: Corey exponent, oil
*n*_*w*_: Corey exponent, water
*P*_*00*_: Dead state pressure, 1 atm = 101,325 Pa (Pa)
*P*_*init*_: Reservoir initial pressure (Pa)
*k*^*0*^_*ro*_: Injected fluid flow rate (m^3^/s)
*S*_*or*_: Residual oil saturation
*S*_*wc*_: Connate-water saturation
*T*_*00*_: Dead state temperature, 25 °C = 298.15 K (K)
*∆P*: Pressure difference (Pa)
*∆T*: Temperature difference (K)
*η*: Pump efficiency
*ρ*_*w*_: Water density (kg/m^3^)
*ρ*_*o*_: Oil density (kg/m^3^)
*φ*: Porosity
μ_o_: Oil viscosity (Pa s)
*μ*_*w*_: Water viscosity (Pa s)
